# Small mammals reduce activity during high moon illumination under risk of predation by introduced predators

**DOI:** 10.1038/s41598-023-37166-1

**Published:** 2023-06-29

**Authors:** P. Taylor, M. Swan, H. Sitters, A. Smith, J. Di Stefano

**Affiliations:** 1 School of Agriculture, Food and Ecosystem Sciences, The University of Melbourne, 4 Water Street, Creswick, VIC 3363 Australia; 2 Present Address: NSW Department of Primary Industries, Vertebrate Pest Research Unit, 1447 Forest Road, Orange, NSW 2800 Australia

**Keywords:** Ecology, Behavioural ecology, Biodiversity

## Abstract

Predation influences prey survival and drives evolution of anti-predator behaviour. Anti-predator strategies by prey are stimulated by direct encounters with predators, but also by exposure to indicators of risk such as moonlight illumination and vegetation cover. Many prey species will suffer increased risk on moonlit nights, but risk may be reduced by the presence of dense vegetation. Determining the role of vegetation in reducing perceived risk is important, especially given predictions of increased global wildfire, which consumes vegetation and increases predation. We used remote cameras in southeastern Australia to compare support for the predation risk and habitat-mediated predation risk hypotheses. We examined the influence of moonlight and understorey cover on seven 20–2500 g mammalian prey species and two introduced predators, red foxes and feral cats. Activity of all prey species reduced by 40–70% with increasing moonlight, while one species (bush rat) reduced activity in response to increasing moonlight more sharply in low compared to high understorey cover. Neither predator responded to moonlight. Our findings supported the predation risk hypothesis and provided limited support for the habitat-mediated predation risk hypothesis. For prey, perceived costs of increased predation risk on moonlit nights outweighed any benefits of a brighter foraging environment.

## Introduction

Predation is a key biological process that influences interactions among individuals, shapes species’ distributions and alters the structure of ecological communities^1,2^. For example, the consumption of prey by predators, and competition between different predator species, can result in downstream influence on other groups of animals and plants, and on the functional roles these species play^1^. Further, the importance of predation to ecosystem function is evidenced by the large effects of predator removal or reintroduction on other species and ecosystem processes^3–5^. The most obvious effects of predation are the direct influence that predators have on prey populations by consuming individuals, but indirect effects are also common. For example, the conservation of mammalian carnivores can increase carbon storage by palatable plants due to the downwards pressure predators place on herbivore populations^6,7^.

From the perspective of prey species, predator activity translates to a perception of risk that varies spatially and temporally, influencing both intraspecific and interspecific interactions^2,8,9^. For example, female song sparrows (*Melospiza melodia*) subject to predator calls but not to actual predation produced 40% fewer offspring per year compared to individuals that were exposed to calls of non-predatory species^10^. Further, in cases where predation has an influence on prey fitness, it can drive the evolution of prey behaviours that minimise detection by predators^2,11^. For instance, optimal foraging theory predicts that prey species trade-off the need to acquire food with the risk of predation associated with foraging^12,13^. To do this prey must be able to evaluate predation risk using either direct or indirect cues^14^. Direct cues include physical encounters, scats, urine, markings and calls, while indirect cues include abiotic factors such as habitat structure, weather (e.g. wind and rain), reproductive status (e.g. breeding or non-breeding) and moonlight^2,14–16^.

Approximately sixty-nine percent of mammals are nocturnal^17^, thus changes in moonlight over the lunar cycle is expected to influence the behaviour of many species^18^. Increased moonlight can improve the hunting efficiency of both mammalian and non-mammalian predators^19,20^, resulting in increased predation risk and reduced activity for prey species^21–24^. For example, the detectability of new holland mice (*Pseudomys navaehollandiae*) decreased with increasing moonlight and six times more survey effort was required to reach 95% confidence that the species was absent during full moon compared to new moon^21^. These observed behavioural patterns form the basis of the predation risk hypothesis, which predicts that increased moonlight will result in lowered activity in prey species and increased activity in predators^25–27^.

Dense vegetation provides mammals with a variety of resources including shelter from predators^20,28,29^. For example, Allenby’s gerbils (*Gerbillus andersoni allenbyi*) exposed to owls and vipers harvested substantially more seed (a measure of perceived predation risk) from sheltered compared to open patches^28^. Potentially, reduced predation risk in sheltered environments can interact with increased predation risk associated with illumination, resulting in the suppressive influence of moonlight on prey activity diminishing with increasing shelter. This interaction is described by the habitat mediated predation risk hypothesis^27^, although tests of this hypothesis have rendered mixed results. For example, consistent with the habitat mediated predation risk hypothesis, Meriam’s kangaroo rat (*Dipodomys merriami*) removed fewer seeds from open habitat compared to sheltered habitat during a full moon^30^. In contrast moonlight had no effect on the use of dense or open microhabitats by the agile antechinus (*Antechinus agilis*)^31^.

In this study, we investigated the response of seven small–medium sized mammals ranging from 20 to 2500 g (prey species) and two introduced predators (feral cats, *Felis catus* and red foxes, *Vulpes vulpes*) to moonlight and vegetation cover in the woodlands of south–western Victoria, Australia. Wildfire is a natural process in this ecosystem, and prescribed fire is used as a management tool to reduce fuel loads and manage biodiversity. Infrequent wildfire and annual application of prescribed fire in our study area has resulted in a large range of vegetation states from open recently burnt sites to dense regenerating areas, making it an ideal location to investigate the responses of prey and predator species to moonlight and vegetation cover. Furthermore, there is increasing evidence that fire-induced vegetation change can increase predation on small mammals by introduced predators^32–34^ thus determining the role of vegetation cover in mediating perceived predation risk is important. For example, increased predation in recently burnt areas with reduced vegetation cover could potentially be moderated by implementing patchy burns resulting in unburnt vegetation within the fire perimeter for prey species to shelter in.

**Figure 1:**
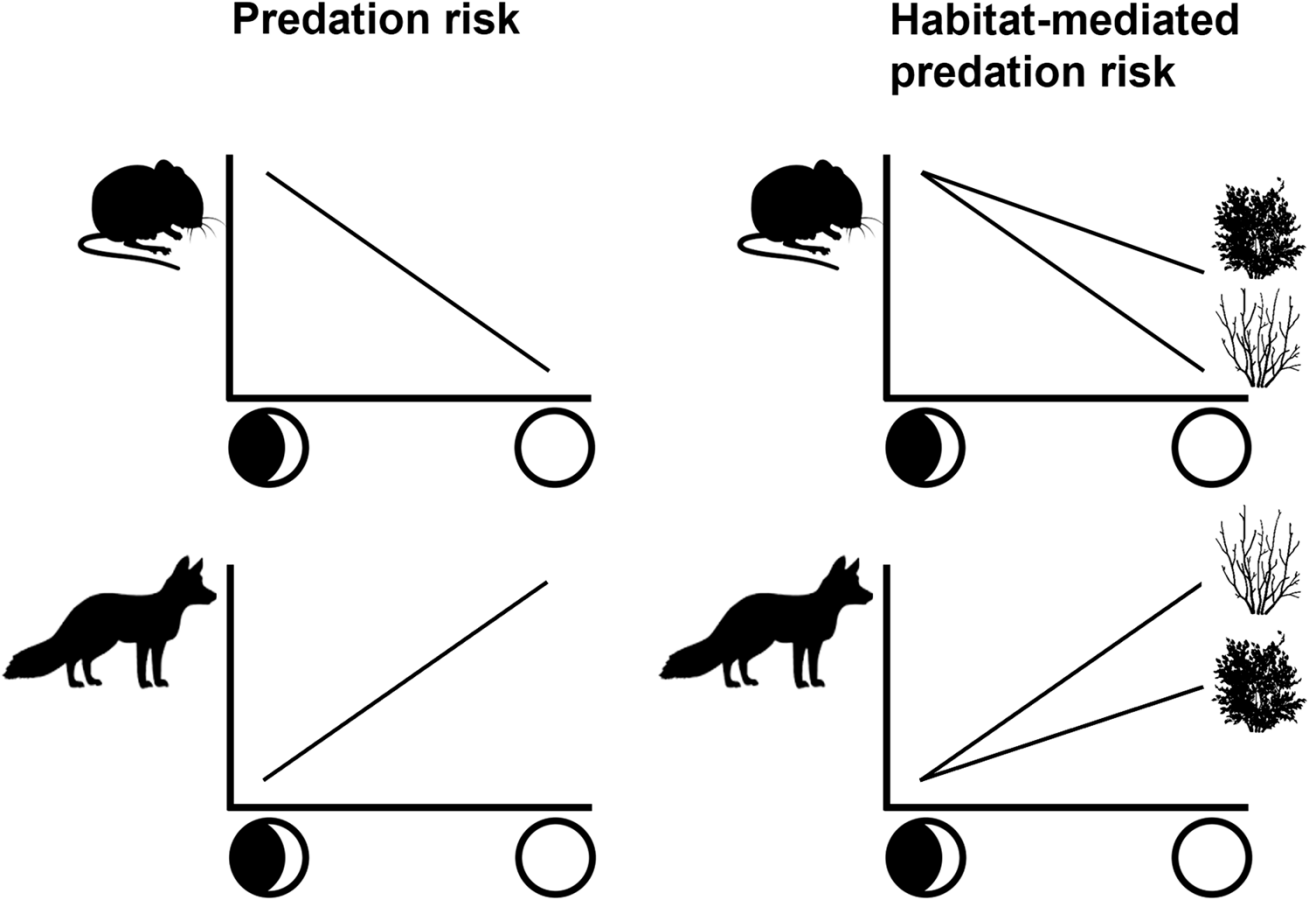
Conceptual model outlining the expected relationships between both prey and predator activity and moonlight under the predation risk and habitat-mediated predation risk hypotheses. The symbols on the left and right of the x-axis represent low and high moon illumination respectively.

We tested the following hypotheses (Fig. 1):

1. *Predation risk hypothesis* Moonlight increases predation risk. We predict a negative relationship between moonlight and prey species activity and a positive relationship between moonlight and predator activity.
2. *Habitat mediated predation risk hypothesis* Moonlight increases predation risk to a greater extent in open compared to sheltered habitats. Both the negative relationship between moonlight and prey activity and the positive relationship between moonlight and predator activity will be stronger in open compared to sheltered habitats.

## Results

We detected 11 native prey species, two introduced prey species and two introduced predator species in 17,699 camera trap nights across 154 sites (Table 1; Supplementary Table S1). Six native prey species (heath mouse, yellow-footed antechinus, bush rat, southern brown bandicoot, common ringtail possum and common brushtail possum) one introduced prey species (house mouse) and two introduced predators (feral cat and red fox) occurred at ≥ 10 sites and were subject to formal analysis (Table 1).

**Table 1:** The number of sites at which each prey and predator species was detected.

Species	Number of sites detected		
	2018/2019	2019/2020	Total
Prey species			
Bush rat (*Rattus fuscipes*)	26	14	29
Common brushtail possum (*Trichosurus vulpecula*)	74	75	82
Common ringtail possum (*Pseudocheirus peregrinus*)	29	33	48
Heath mouse (*Pseudomys shortridgei*)	34	33	41
House mouse (*Mus musculus*)	22	11	29
Southern brown bandicoot (*Isoodon obesulus*)	10	12	14
Yellow-footed antechinus (*Antechinus flavipes)*	37	30	51
Introduced predators			
Feral cat (*Felis catus*)	6	8	12
Red fox (*Vulpes vulpes*)	39	42	69

The first two numeric columns represent the number sites occupied during the 2018/2019 and 2019/2020 sampling seasons while the last column represents the total number of uniquely occupied sites across both seasons. Only species recorded at ≥ 10 sites in total are included; species detected at fewer sites are shown in Supplementary Table S1.

**Figure 2:**
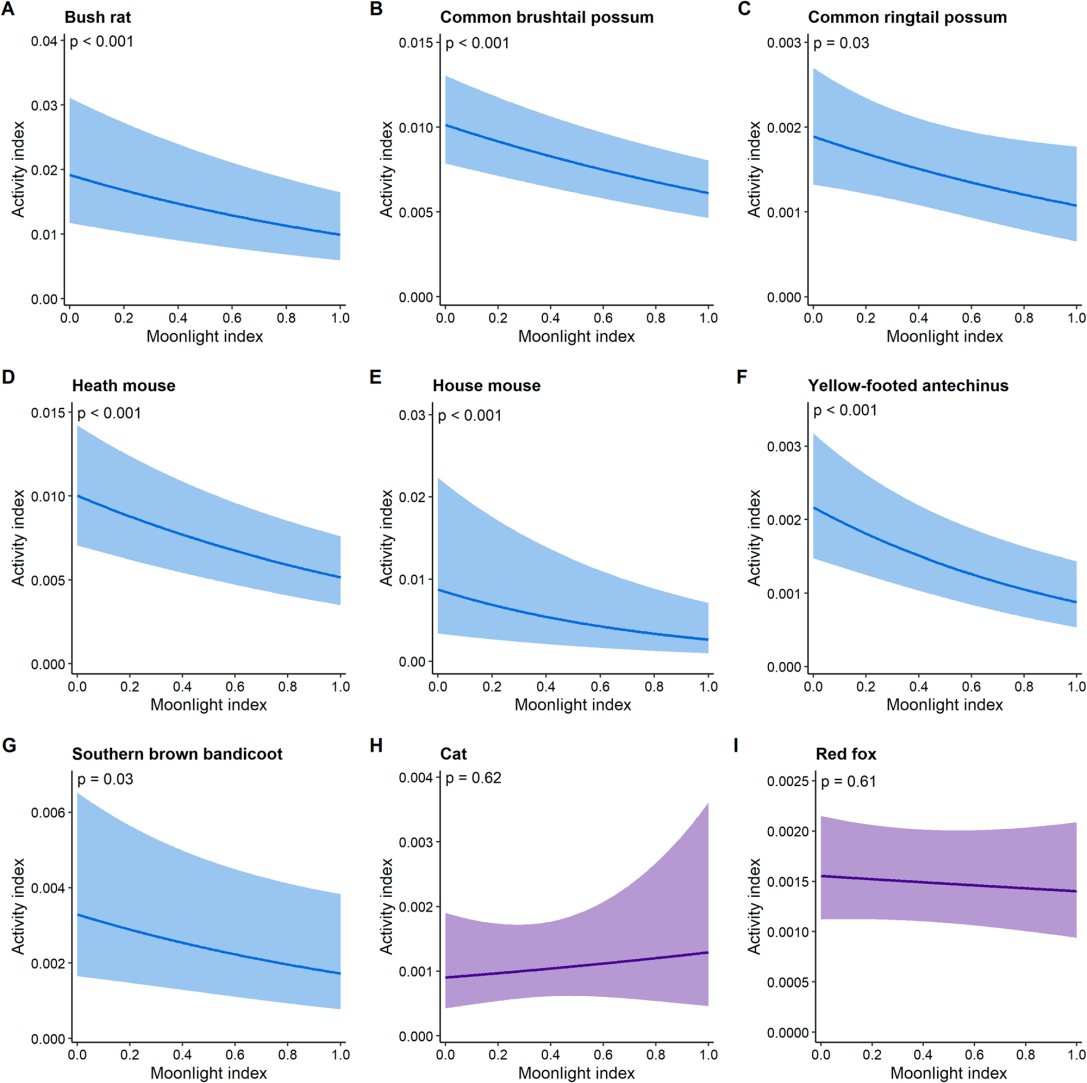
Predictions from a generalised linear mixed model representing the responses of prey and predator species to the moonlight index. Detectable negative relationships (blue) indicate support for the predation risk hypothesis. Null effects are shown in purple. If cloud cover or season were present, predictions were generated at the mean of cloud cover and for season 1. Shading reflects 95% confidence limits.

Among prey species, our analysis demonstrated strong support for the predation risk hypothesis; increased moonlight resulted in reduced activity for every species (Fig. 2, Table 2). Declines in activity were substantial and ranged between 40% for the common brushtail possum and 70% for the house mouse (Fig. 2).

**Table 2:** Models of the response of prey and predator species to the moonlight index (MI), understorey cover (UC), cloud cover (C) and season (S).

Species	Model	Estimate (95% CL)	Supported hypothesis	Change in AICc	Akaike weight	R^2^m (R^2^c)
Bush Rat (*Rattus fuscipes*)	MI × UC + C + S	0.20 (0.10, 0.30)	HMPR	0.00	1.00	0.21 (0.53)
	MI + UC + C + S	− 0.22 (− 0.29, − 0.16)	PR	13.07	0.00	0.18 (0.51)
	Null			305.03	0.00	0.00 (0.47)
Common Brushtail Possum (*Trichosurus vulpecula*)	MI + C + S	− 0.17 (− 0.22, − 0.12)	PR	0.00	0.52	0.01 (0.31)
	MI × UC + S + C	− 0.04 (− 0.10, 0.01)		1.23	0.28	0.01 (0.31)
	MI + UC + S + C			1.99	0.19	0.01 (0.31)
	Null			63.87	0.00	0.00 (0.30)
Common Ringtail Possum (*Pseudocheirus peregrinus*)	MI	− 0.19 (− 0.36, − 0.01)	PR	0.00	0.25	0.01 (0.23)
	MI + S			1.44	0.12	0.01 (0.24)
	MI + C			1.98	0.09	0.01 (0.23)
	MI + UC			2.00	0.09	0.01 (0.23)
	MI × UC	0.12 (− 0.05, 0.29)		2.02	0.09	0.01 (0.24)
	Null			2.72	0.07	0.00 (0.23)
Heath Mouse (*Pseudomys shortridgei*)	MI + UC + C	− 0.22 (− 0.30, − 0.14)	PR	0.00	0.32	0.03 (0.36)
	MI + UC + C + S			0.47	0.25	0.04 (0.36)
	MI × UC + C	− 0.04 (− 0.13, 0.04)		1.07	0.18	0.03 (0.35)
	MI × UC + C + S			1.53	0.15	0.03 (0.35)
	Null			51.98	0.00	0.00 (0.35)
House Mouse (*Mus musculus*)	MI + UC + S	− 2.28 (− 2.91, − 1.64)	PR	0.00	0.52	0.31 (0.70)
	MI × UC + S	− 0.04 (− 0.21, 0.14)		1.85	0.21	0.32 (070)
	MI + UC + S + C			1.97	0.20	0.31 (0.70)
	Null			217.24	0.00	0.00 (0.31)
Yellow-footed Antechinus (*Antchinus flavipes*)	MI × UC + C	− 0.10 (− 0.23, 0.03)		0.00	0.32	0.05 (0.33)
	MI + UC + C	− 0.30 (− 0.44, − 0.16)	PR	0.18	0.30	0.06 (0.34)
	MI × UC + C + S			1.68	0.14	0.05 (0.34)
	MI + UC + C + S			1.89	0.13	0.06 (0.34)
	Null			27.04	0.00	0.00 (0.32)
Southern Brown Bandicoot (*Isoodon obesulus*)	MI + UC + S	− 0.21 (− 0.41, − 0.02)	PR	0.00	0.25	0.14 (0.37)
	MI × UC + S	0.16 (− 0.15, 0.47)		0.94	0.16	0.17 (0.39)
	MI + UC			1.32	0.13	0.12 (0.35)
	MI + UC + S + C			1.57	0.11	0.14 (0.37)
	Null			20.85	0.00	0.00 (0.34)
Feral cat (*Felis catus*)	Null		None	0.00	0.18	0.00
	UC + C			0.73	0.13	0.07
	UC			0.77	0.13	0.02
	MI + C	0.12 (− 0.35, 0.59)		1.70	0.08	0.05
	MI			1.81	0.07	0.00
	MI × UC + C	0.20 (− 0.29, 0.69)		3.84	0.03	0.09
Red fox (*Vulpes vulpes*)	UC + S		None	0.00	0.37	0.02 (0.24)
	UC + S + C			1.64	0.17	0.02 (0.24)
	MI + UC + S	− 0.03 (− 0.16, 0.10)		1.73	0.16	0.02 (0.24)
	MI × UC + S	− 0.02 (− 0.15, 0.11)		3.66	0.06	0.02 (0.24)
	Null			7.74	0.01	0.00 (0.23)

Models were ranked using Akaike’s information criterion adjusted for small sample size (AICc) and those displayed include all models within 2 AICc units of the best and, if not already in this set, the best models containing the moonlight index with and without its interaction with understorey cover. The null model is also included for reference. Estimates and their 95% confidence limits have been included for the MI term in the most highly ranked model containing the moonlight index (a test of the predation risk hypothesis) and the MI by UC interaction term for the most highly ranked model containing the interaction between the moonlight index and understorey cover (a test of the habitat mediated predation risk hypothesis). Hypotheses that are supported by the data are indicated in the Supported hypothesis column; habitat mediated predation risk and predation risk are coded as HMPR and PR. Change in AICc represents the difference in AICc between the associated model and the best model, Akaike weights are the probability that the model is the best in the set of tested models, and marginal and conditional r-squared values (R^2^m and R^2^c) represent the variance explained by fixed factors and both fixed and random factors respectively. R^2^c associated with cat models do not exist as random effects were excluded (see methods for details).

**Figure 3:**
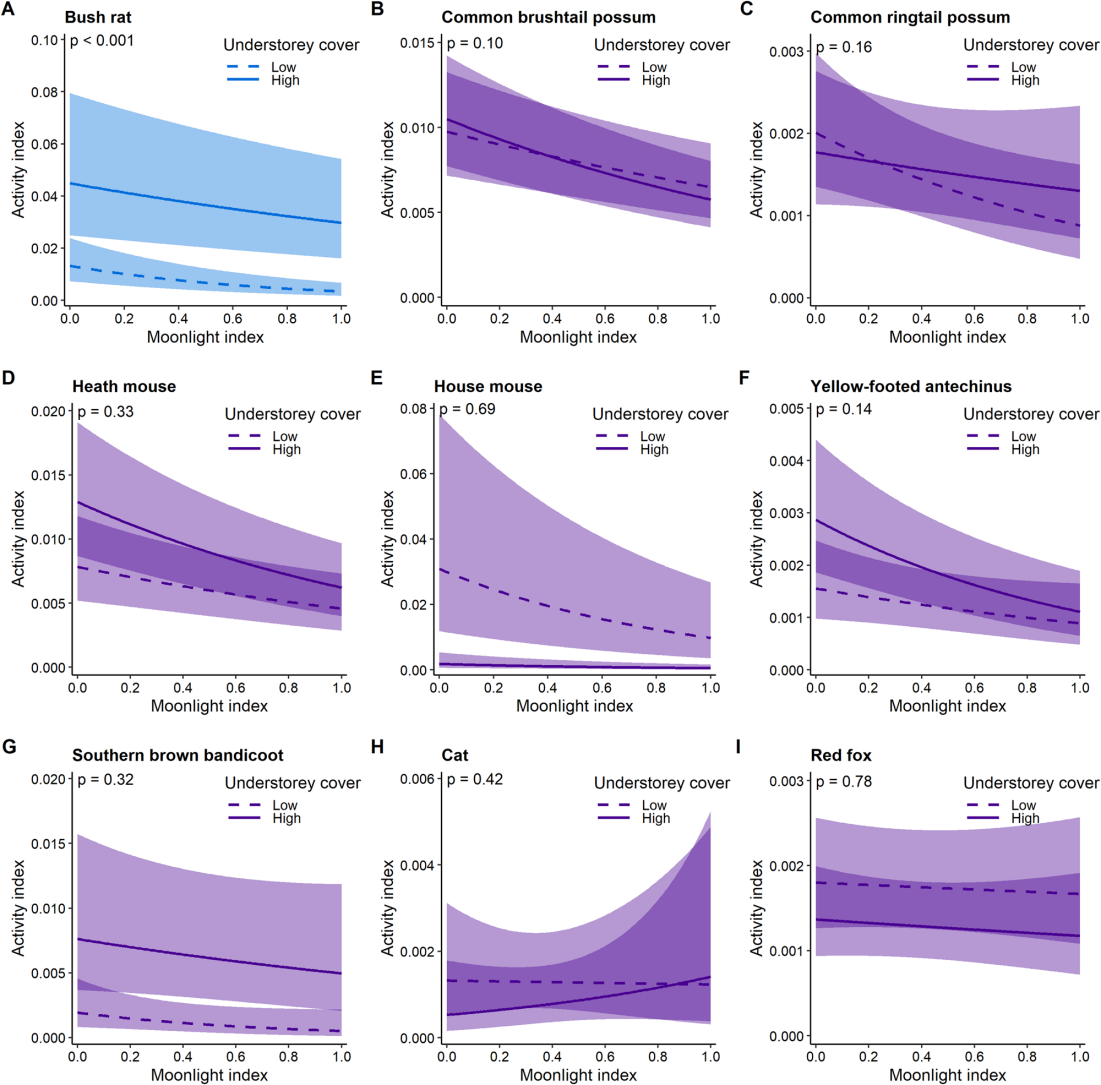
Predictions from a generalised linear mixed model representing the responses of prey and predator species to the interaction between the moonlight index and understorey cover; species’ responses to the moonlight index are shown at low (25th percentile) and high (75^th^ percentile) understorey cover. Detectible interaction effects (blue) indicate support for the habitat mediated predation risk hypothesis. Null effects (i.e., no detectible interaction) are shown in purple. If cloud cover or season were present, predictions were generated at the mean of cloud cover and for season 1. Shading reflects 95% confidence limits.

In contrast, there was little support for the habitat-mediated predation risk hypothesis with bush rats the only species for which an interaction between the moonlight index and understorey cover was detected (Fig. 3, Table 2). When understorey cover was high (75th percentile), increased moonlight resulted in a 34% decline in bush rat activity, while the decline at low understorey (25th percentile) cover was 75% (Fig. 3).

Neither of the predators, feral cats and red foxes, responded either positively or negatively to moonlight (Fig. 2, Table 2). Cats did not respond to any of our modelled predictors (the null model was the best), while for foxes we detected a negative response to understorey cover (estimate (95% CL) − 0.23 (− 0.45, − 0.01), *p* = 0.04).

For all modelled species, the estimate and lower and upper 95% confidence limits associated with each parameter in the top models are presented in Supplementary Tables S2–S10.

## Discussion

Prey species have evolved to respond to both direct and indirect cues of predation risk but can also learn appropriate responses to novel threats over shorter time periods^14,35^. In the absence of direct cues, prey species may change their behaviour in response to stimuli that indirectly indicates an increase in predation risk, such as moonlight and vegetation cover, helping to decrease interactions with predators^27^. We found that the predation risk hypothesis was most strongly supported by our results; the activity of all seven prey species was negatively correlated with the moonlight index. The response of only one prey species, the bush rat (*Rattus fuscipes*), supported the habitat mediated predation risk hypothesis while cats and foxes did not respond to the moonlight index. We discuss our results in relation to the key hypotheses and consider their implications for land management.

### Predation risk hypothesis

There was strong support for the predation risk hypothesis, with all six native species and the introduced house mouse reducing their activity with increasing moonlight. Our results are consistent with a recent global meta-analysis showing that, on average, moonlight suppressed the activity of mammalian prey that use non-visual (e.g. olfactory) senses to detect predators^27^. Nevertheless, support for the hypothesis is not universal. For example, while Bolam’s mouse (*Pseudomys bolamii*), mallee ningaui (*Ningaui. yvonneae*) and verreaux mouse (*Praomys verreauxii)* all responded negatively to moonlight^15,23^, several critical weight range (35–5500 g)^36^ prey species in a fenced conservation reserve in southeastern Australia did not respond to moonlight^37^, presumably because introduced vertebrate predators were absent.

Considering variable support in the literature for the predation risk hypothesis, the strong negative response to moonlight shown by all prey species in our study suggests that predation risk could be substantial. Further, several lines of evidence point to much of this risk originating from introduced vertebrate predators. First, foxes were widespread in our study area (Table 1) and cats were also detected but at fewer sites. Although we do not have good evidence of fox and cat densities, our sites were among a matrix of pasture and plantations with an extensive track network—factors that may increase predation pressure by these species^38,39^. Second, native dingoes are absent from western Victoria^40^, and as they can supress cat and fox populations^41,42^, their absence is likely to intensify predation pressure by these two introduced species. Finally, as noted above, a fenced community of prey species did not respond to moonlight when introduced predators were excluded^37^ . Nevertheless, our prey species are also subject to predation by two nocturnal foragers (Barn Owl, *Tyto alba* and Southern Boobook, *Ninox boobook*) who likely influence prey behaviour to some extent. Determining the relative influence of native and introduced predators on anti-predator behaviour is a fertile area for future work; however, manipulative experiments are required to disentangle these effects.

Our findings suggest that native prey are actvating a strong anti-predator response strategy in the face of predation risk from by introduced species. This is consistent with recent evidence demonstrating that responses of prey to native and introduced predators are usually similar, and even if prey are initially naïve to novel predators they quickly learn apropriate anti-predator strategies over short time frames^35,43,44^. Our results are in contrast to the prey naivety hypothesis that predicts native prey will fail to recognise and respond appropriately to an evolutionary novel threat^45,46^. The strong negative response to moonlight by all prey in this study support the view that prey are susceptible to threats from all predators regardless of their origins, and that the strength of anti-predator responses are more likely to be driven by other factors, such as the relative mass of predators and prey^43^.

Predictions of increased predator activity with moonlight under the predation risk hypothesis were not realised; the moonlight index was not associated with the activity of foxes or cats. Previous studies from diverse locations have reported an increase in hunting success or activity with moonlight for both species^19,47,48^. However, one Australian study found that foxes ate fewer small mammals when the moon was full compared to other phases of the lunar cycle^49^. There may be several reasons why the predators in our study did not respond positively to moonlight. One possibility is that the negative association between prey species activity and moonlight acted as an effective anti-predator strategy and increasing their activity when moonlight was high rendered no benefit to predators. Red foxes and cats eat a wide variety of food items^50,51^, so it is possible that their foraging strategies shift when small mammal prey species are less active when the moon is bright. For example, Jaguars changed their habitat use during the full moon when armadillos avoided foraging above ground^52^. Alternatively, a lack of numerical response (more predators or more active predators) may be counterbalanced by a functional response, (i.e. hunting efficiency is greater without an increase in predator activity)^34^. In general, the optimal foraging decisions for medium-sized predators are likely to be less predictable than for small mammals. Medium-sized predators are at risk of predation themselves, so the optimal behavioural pattern involves complex trade-offs between foraging and predator avoidance^53^.

### Habitat-mediated predation risk hypothesis

Although many studies have analysed how moonlight illumination affects small mammal behaviour, few have extended their analysis to investigate the interaction between moonlight and vegetation cover. We found limited support for the habitat mediated predation risk hypothesis, with only bush rats showing a smaller decline of activity with increased moonlight at high compared to low vegetation cover. This suggests that bush rats perceived an elevated predation risk in more open vegetation compared to closed vegetation when moonlight illumination was high. The overall scarcity of interactions between moonlight and vegetation cover suggests dense understorey vegetation does not substantially alter perceived predation risk at high moonlight for most of the prey species we studied.

Compared to the predation risk hypothesis there are fewer tests of the habitat mediated version, and existing results provide variable support. For example, in a meta-analysis of giving up density (GUD) studies, seed consumption increased on dark compared to moonlit nights, but this effect was similar in open and closed habitats^27^. Studies using different response variables have rendered similar results; for example, the number of captures of agile antechinus (*A. agilis*) in open and closed habitats was not influenced by increasing moonlight or experimentally modified light levels^31^. In contrast, desert rodents in China and the house mouse (*Mus domesticus*) in Australia shifted to foraging in denser patches of vegetation when moon illumination was high compared to when it was low, providing support for the habitat-mediated predation risk hypothesis^54,55^.

Four prey species (bush rat, yellow-footed antechinus, heath mouse and southern brown bandicoot) showed increased activity in dense compared to open understorey vegetation regardless of moonlight effects. This is a common response for many small mammals; sheltered habitats may reduce predation risk as they provide greater refuge from predators than open areas^56–58^. One notable exception was the house mouse whose activity was substantially higher where the understorey was more open. This species is introduced and known to occupy open or disturbed environments, which may be a strategy to avoid competition with native small mammals in Australian systems^59^. Foxes also responded negatively to understorey cover, likely reflecting their attraction to recently burnt areas^32,33^.

### Cloud cover

We expected cloud cover to have a positive influence on prey species activity levels as it may limit the amount of moonlight reaching the forest floor, reducing the suppressive influence of moon illumination. Cloud cover had a positive influence on the activity of three prey species, a negative influence on the activity of one prey species, and did not influence the activity of the remaining three prey species or the two predators. Many studies on the effect of moonlight on prey species have failed to incorporate cloud cover into their analyses^24,30,60^. Those that have exhibit variable outcomes similar to our observations. For example, in southeastern Australia, prey species increased their activity during periods of high cloud cover^37^. In contrast, cloud cover was found to have no significant effect on the response of giant kangaroo rats to moonlight^61^. One explanation for these mixed results is that weather station data do not include metrics of cloud thickness, a factor that likely influences moon illumination.

Furthermore, cloud is related to other weather factors that may affect mammal behaviour, independent of their effects on moonlight, such as atmospheric pressure, precipitation and wind. Both wind and precipitation may influence predation risk by interfering with the auditory and olfactory cues of either predator or prey. For example, high wind speed has been found to have a positive influence on capture rates of small mammals as the wind noise may mask their movement, decreasing predation risk^15^. Disentangling the interacting influences of weather, cloud cover and moon illumination is challenging and will require manipulative experiments to tease apart their effects.

## Conclusion

The activity of all six native mammal prey species and the introduced house mouse was negatively associated with moonlight levels, lending strong support to the predation risk hypothesis. In addition, four native prey species (bush rat, yellow-footed antechinus, heath mouse and southern brown bandicoot) were more active at higher compared to lower vegetation cover. Predation, especially from introduced predators, has been a key driver of mammal extinction in Australia and other parts of the world^62^ and maintaining habitats containing dense vegetation is seen as a key conservation action to protect vulnerable native prey. This is especially pertinent in flammable ecosystems; for example in Australia there is evidence highlighting increased vulnerability of prey species to introduced cats and foxes immediately after fire, with patchy fires that leave unburnt vegetation as shelter suggested as a potential conservation strategy reviewed by^34^. It remains unknown, however, how moonlight interacts with fire and vegetation to influence the vulnerability of native species to predation. For instance, the vulnerability of native prey species to predation occurring in the days to weeks after prescribed fire could potentially be reduced if prescribed burns were conducted at an appropriate point in the lunar cycle; however further research is needed to test this hypothesis.

## Methods

### Study area

**Figure 4:**
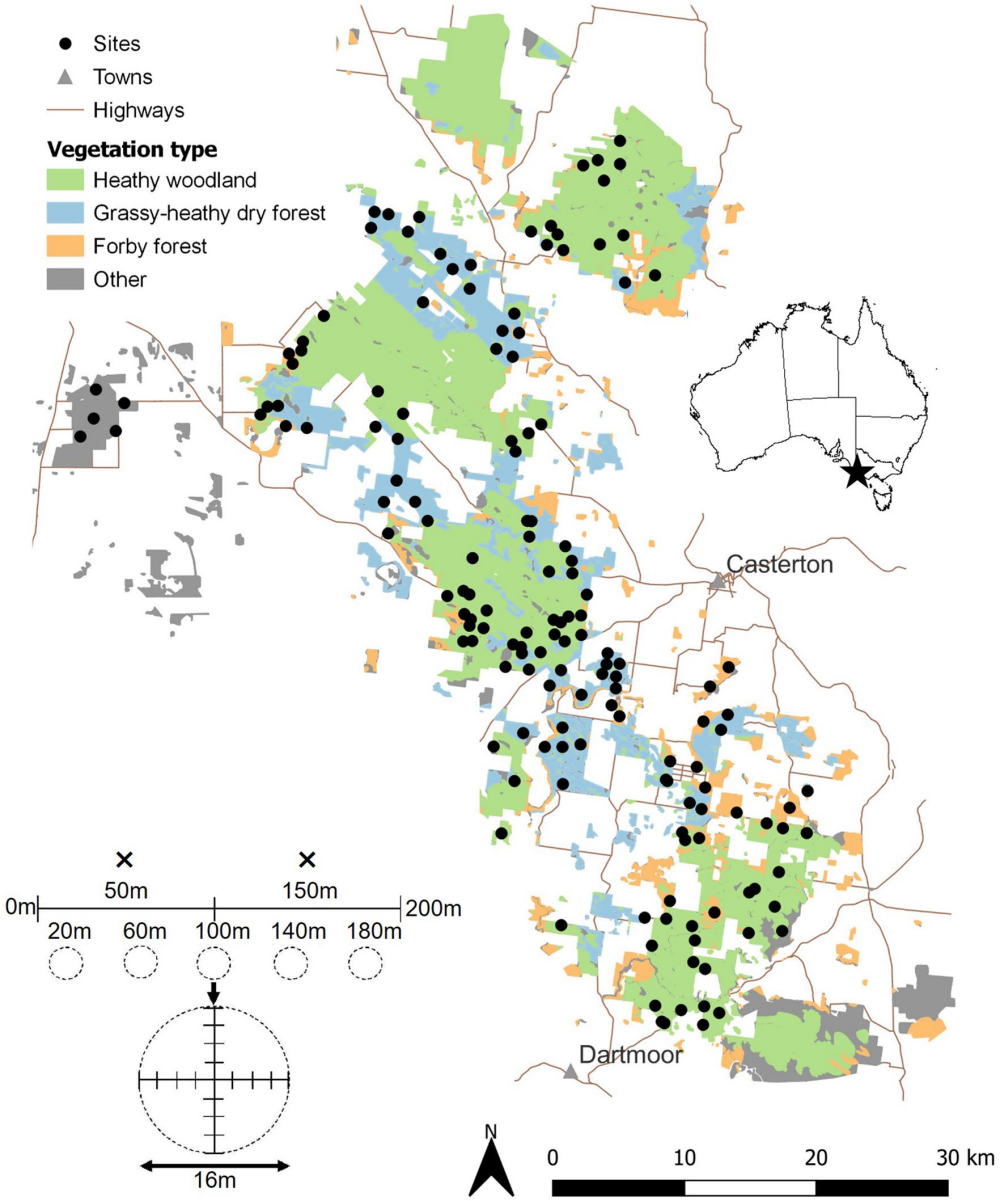
Study area map with sites overlying the main vegetarian types in southeastern Australia (see inset at the top-right). The inset at the bottom-left shows the location of the cameras (crosses) and vegetation sampling plots (circles) along a 200 m transect. An enlargement of a vegetation sampling plot is also shown.

The study area (Fig. 4) has a temperate climate characterised by cool, wet winters and warm dry summers. Mean annual minimum and maximum temperatures are 8.3 °C and 20.0 °C respectively while mean annual rainfall ranges from 656 mm in the north to 741 mm in the south^63^. Mean annual minimum and maximum temperatures are 8.3 °C and 20.0 °C respectively while mean annual rainfall ranges from 656 mm in the north to 741 mm in the south^63^. There is little topographical variation (70–90 m a.s.l) and soils are deep, highly acidic, fine to medium-grained Aeolian quartz sand with little organic matter^64^.

Large patches of native eucalypt woodland and forest (1000–10,000 ha) are surrounded by pasture and plantation forestry. The native vegetation is primarily composed of Heathy woodland, Grassy/heathy dry forest and Forby forest. Heathy woodland is characterised by shrubs, including silver banksia (*Banksia marginata*) and heath tea tree (*Leptospermum myrsinoides*), and the grass trees *Xanthorrhoea australis* and *X. caespitosa.* It has an overstorey of brown stringybark (*Eucalyptus baxterii*) but is treeless in low-lying areas with impeded drainage^33^. Grassy/heathy dry forest has an understorey of bracken (*Pteridium esculentum*), sedges, grass trees (*X. australis*) while brown stringybark dominates the overstorey. Forby forest has an open understorey dominated by bracken, sedges and tussock grasses. Black wattle (*Acacia mearnsii*) occurs through the sparse midstory while messmate (*E. obliqua*) and brown stringybark dominate the overstorey. Climatic and soil gradients result in taller and shrubbier woodlands in the south. The region is susceptible to wildfires and planned burns are applied regularly (usually during autumn) to reduce wildfire risk and promote biodiversity.

### Study design

To ensure we sampled a range of vegetation densities, we stratified the study area by (a) vegetation type (described above) and (b) fire age class, a categorical representation of time since fire, defined as recently burnt (0–3 years), early successional (4–10 years), mid successional (11–34 years) and late successional (35+ years)^65^. Within these strata we randomly selected a total of 154 sites in loose clusters of 5 to increase sampling efficiency. Sites were a minimum of 1 km from each other and at least 50 m from roads and other edges created by vegetation types or age classes. At each site we established a 200 m randomly-orientated transect. Native vegetation and fire history maps used during the design phase were accessed from the Data Vic website (https://www.data.vic.gov.au/).

### Camera surveys

Prey and predator activity were detected using motion-sensing cameras. Camera trapping took place principally during the austral spring, summer and autumn; November to June in 2018/2019 and repeated November to April in 2019/2020. Camera trapping was conducted with approval from the University of Melbourne Animal Ethics Committee (ID 1604071) and a scientific research permit issued by the Department of Environment, Land, Water and Planning (ID 10008227).

One ReconyxTM HC500 (infrared) and one ReconyxTM HC550 (white-flash) was placed at each site at the 50 m and 150 m (randomly allocated) mark of the transect (Fig. 4). The white-flash cameras were included to help identify small mammals where colour provides diagnostic information. Cameras were attached to trees 30 cm off the ground, facing a bait station 1.5 m to the south. The bait consisted of a mixture of rolled oats, peanut butter, golden syrup and pistachio essence and was placed into perforated polypipe canisters (10 × 5 cm) attached to wooden stakes and suspended 20 cm off the ground (Supplementary Fig. S1). Vegetation was cleared between the camera and bait as well as 50 cm behind and to the sides of the bait allowing for better species identifications and false trigger reduction. Cameras were set on high sensitivity and five images were taken in rapid succession for each activation with no delay between activations. Resolution was set to 3.1 MP and night mode was balanced. Cameras were deployed for 30 days to capture the duration of the lunar cycle. Of 616 deployments (154 sites × 2 camera × 2 seasons), 44 cameras failed but 26 of these were successfully redeployed for the full 30 days. Deployment time for the remaining 18 cameras ranged between 0 and 29 days. We excluded one site in season 2 as both cameras failed. At all other sites at least one camera operated for the full 30-day deployment period.

Five researchers identified camera images in both 2018/2019 and 2019/2020. Species were identified using a field guide^66^ and reference images from previous studies. Positively identified images were labelled with a species code using digiKam version 6.2.0 (www.digikam.org/) and we extracted and summarised the metadata in R version 4.0.2 (R Core Team, 2020) using camtrapR^67^. These data were converted into an activity index representing the number of hours per night each species was detected; we did not calculate activity for species occurring at fewer than 10 sites (Table S1). The activity index was calculated separately for each night of the 30-day deployment period to reflect changes in activity across the lunar cycle.

### Vegetation surveys

Vegetation surveys were conducted at each site between October 2019 and March 2020 at the 20 m, 60 m, 100 m, 140 m and 180 m marks of each transect (Fig. 4). At each of these five locations, a 2 m pole was placed at 16 sampling points to quantify presence or absence of vegetation between 0 and 100 cm (a total of 80 sampling points per site). The number of presences out of 80 was used as an understorey cover score for statistical modelling. We used vegetation within 100 cm of the ground as preliminary analysis of vegetation data sampled between the ground and the canopy (unpublished data) showed that on average 72% of the vegetation resource was within this zone.

### Moon illumination

Proportion of the moon disc illuminated, moonrise and moonset times and sunrise and sunset times for each day during the study period were calculated using the R package suncalc^68^. Moon illumination was calculated at Casterton (37.58 S, 141.4 E; centrally located in the study area) as the proportion of the moon disc illuminated, multiplied by the proportion of the night that the moon was above the horizon. The resulting index ranged from 0 to 1 with 0 representing a new moon or a night when the moon does not appear above the horizon, and 1 representing a full moon that is above the horizon for the entire night.

### Cloud cover

Cloud cover data were acquired from three weather stations within 100 km of the study area; Mount Gambier Aero, Portland Airport and Hamilton (Cashmore Airport)^63^. Cloud cover was measured as the fraction of the total sky covered in cloud in eighths. At each station cloud cover values at 2100, 0000 and 0300 h each night were averaged, and then the values from the three stations were averaged to represent nightly cloud cover for the whole study area. Cloud cover ranged from 0 to 8 where 0 represents the absence of clouds and 8 represents a completely overcast night.

### Statistical modelling

Models were built in R version 4.2.0^69^ to determine the response of species activity to the moonlight index, understorey cover and cloud cover. We also included season to account for the surveys in 2018/2019 and 2019/2020. Although we used vegetation type and fire age class as strata in our design, we did not use these variables for modelling. We excluded vegetation type as heathy woodland dominated the study area thus the number of sites in other vegetation types was small (Fig. 4). We excluded fire age class (or time since fire) as the reason for selecting sites within different age classes was to ensure we sampled a broad range of understorey cover, which we then used as a predictor variable. Prior to analysis, the correlation between the moonlight index, understorey cover and cloud cover was checked using scatterplots and Pearson’s correlation coefficient; all correlations were ≤ |0.20| indicating that collinearity was acceptable for modelling^70^. Predictor variables were centred and standardised to allow for better interpretation of interactions between continuous variables and to make coefficients associated with different predictor variables comparable^71^. Data for modelling was derived from sites where species were present, and only species occurring at ≥ 10 sites were included.

We used a Generalised Linear Mixed Model (GLMM) with a binomial family to model species activity as a proportion; the number of hours each species was detected per night divided by the number of hours in the night (defined as the number of complete hours after sunset and before dawn). The response variable was constructed in this way to account for variation in night length during the study period (November to June in season 1 and November to April in season 2). We checked for overdispersion using the performance package^72^; the dispersion ratio was acceptable (< 1.40) in all cases with the exception of house mouse models where the maximum value was 2.0. We modelled this species using a beta-binomial family to resolve this problem^73^. Camera nested within site (site/camera) was used as the random factor to account for two sources of non-independence; the use of two cameras per site and the repeated sampling of each site in two seasons. In cat models we excluded the random part of the model as the variance associated with it was zero. We did not use models that accounted for imperfect detection, however previous work in study area suggests that our camera deployment period would result in very high detection probability for our suite of species^74^.

We used the package glmmTMB^75^ to build 17 models per species to test predictions associated with the predation risk and habitat mediated predation risk hypotheses (Fig. 1). The core model testing the predation risk hypothesis included the moonlight index as the sole predictor, and the core model testing the habitat mediated predation risk hypothesis included the moonlight index, understorey cover and their interaction. For each of these base models we built additional models that included the additive effects of (1) cloud cover, (2) season and (3) both cloud and season (eight models in total). We created an additional four models where the moonlight index was replaced with understorey cover, and another four that included only the additive effects of moonlight and understory cover (i.e. without an interaction term). We also included a null model as a point of reference. A full list of models and their purpose can be found in Supplementary Table S11.

Models were ranked using the small sample size correction of Akaike’s Information Criterion (AICc) computed in the package MuMIn^76^ with lower AICc values representing higher ranked, more parsimonious models. Akaike weights, the probability that a model is the best in the set, were also calculated. For each species we used the highest-ranked model including the moonlight index (but without the interaction between moonlight and understorey vegetation) to test the predation risk hypothesis and the highest-ranked model including the interaction between the moonlight index and understorey vegetation to test the habitat-mediated predation risk hypothesis. Predictions from these two models were represented graphically using the packages ggplot2^77^ and cowplot^78^. For some species, several models received similar support, so we tabulated all models within 2 AICc units of the best and calculated and marginal and conditional r-squared values (R^2^m and R^2^c, respectively) using the package performance^72^. R^2^m represents the variance explained by the fixed effects only while R^2^c represents the variance explained by both fixed and random effects.

## Supplementary Information

Supplementary Information.

## Data Availability

The datasets generated and analysed during the current study are available in the Figshare repository, https://figshare.com/articles/dataset/Moonlight_and_predation_risk/21861846.
