# Supplementary material for: Small mammals reduce activity during high moon illumination under risk of predation by introduced predators
Source: Sci Rep. 2023 Jun 29;13:10532. doi: 10.1038/s41598-023-37166-1 (PMC10310734; doi:10.1038/s41598-023-37166-1)
Supplement: Supplementary file 1 — Supplementary Information. [file 41598_2023_37166_MOESM1_ESM.pdf]

Supplementary Information

Small mammals reduce activity during high moon illumination under risk of predation by introduced predators

Taylor, P[1,2]., Swan, M[1]*., Sitters, H[1]., Smith, A[1]., & Di Stefano, J[1].

[1] School of Ecosystem and Forest Sciences, the University of Melbourne. 4 Water Street, Creswick VIC 3363, Australia.

[2] Current address: NSW Department of Primary Industries, Vertebrate Pest Research Unit, 1447 Forest Road, Orange, NSW 2800, Australia.

*Corresponding author: swanm@unimelb.edu.au

Supplementary Table S1. List of species recorded at too few sites for modelling including the number of sites each species was recorded at in 2018/2019 and 2019/2020 field seasons and total number of sites mammal species were recorded at over both seasons.

**Species**	**Number of sites detected**		
	2018/2019	2019/2020	Total
Agile antechnius (*Antechinus agilis)*	0	1	1
Black rat (*Rattus rattus*)	4	2	4
Long-nosed potoroo (*Potorous tridactylus*)	1	4	4
Silky mouse (*Pseudomys apodemoides*)	1	3	3
Swamp antechnius (*Antechnius minimus*)	7	6	8
Swamp rat (*Rattus lutreolus*)	4	2	5

Supplementary Tables S2-S10. Models of the response of prey and predator species to the moonlight index (MI), understorey cover (UC), cloud cover (C) and season (S). Models were ranked using Akaike's information criterion adjusted for small sample size (AICc) and those displayed include all models within 2 AICc units of the best and, if not already in this set, the best models containing the moonlight index with and without its interaction with understorey cover. The null model is also included for reference. Estimates and lower and upper confidence limits (CL) are derived from generalised linear mixed effects models run on standardised and centred variables.

Supplementary Table S2. Bush Rat (*Rattus fuscipes*). The negative estimate for season reflects higher activity in season 1 (2018/19) compared to season 2 (2019/20).

Model	Parameter	Estimate	Lower CL	Upper CL	P-value	Change in AICc	Akaike weight
MI × UC + C + S						0.00	1.00
	Moonlight index	-0.34	-0.44	-0.25	<0.001		
	Understorey cover	1.00	0.55	1.45	<0.001		
	Moonlight Index:Understorey cover	0.20	0.10	0.30	<0.001		
	Cloud	0.24	0.17	0.32	<0.001		
	Season	-0.90	-1.04	-0.76	<0.001		
MI + UC + C + S						13.07	0.00
	Moonlight index	-0.22	-0.29	-0.16	<0.001		
	Understorey cover	0.93	0.49	1.38	<0.001		
	Cloud	0.24	0.17	0.31	<0.001		
	Season	-0.91	-1.04	-0.77	<0.001		
Null						305.03	0.00

Supplementary Table S3. Common Brushtail Possum (*Trichosurus vulpecula*). The positive estimate for season reflects lower activity in season 1 (2018/19) compared to season 2 (2019/20).

Model	Parameter	Estimate	Lower CL	Upper CL	P-value	Change in AICc	Akaike weight
MI + C + S						0.00	0.52
	Moonlight index	-0.17	-0.22	-0.12	<0.001		
	Cloud	-0.09	-0.14	-0.04	<0.001		
	Season	0.23	0.13	0.33	<0.001		
MI × UC + C + S						1.23	0.28
	Moonlight index	-0.17	-0.22	-0.12	<0.001		
	Understorey cover	0.01	-0.23	0.24	0.96		
	Moonlight Index:Understorey cover	-0.04	-0.10	0.01	0.10		
	Cloud	-0.09	-0.14	-0.04	<0.001		
	Season	0.23	0.13	0.33	<0.001		
MI + UC + C + S						1.99	0.19
	Moonlight index	-0.17	-0.22	-0.12	<0.001		
	Understorey cover	0.01	-0.22	0.24	0.92		
	Cloud	-0.09	-0.14	-0.04	<0.001		
	Season	0.23	0.13	0.33	<0.001		
Null						63.87	0.00

Supplementary Table S4. Common Ringtail Possum (*Pseudocheirus peregrinus*). The positive estimate for season reflects lower activity in season 1 (2018/19) compared to season 2 (2019/20).

Model	Parameter	Estimate	Lower CL	Upper CL	P-value	Change in AICc	Akaike weight
MI						0.00	0.25
	Moonlight index	-0.19	-0.36	-0.02	0.03		
MI + S						1.44	0.12
	Moonlight index	-0.19	-0.36	-0.02	0.03		
	Season	0.12	-0.20	0.45	0.45		
						1.98	0.09
MI + C	Moonlight index	-0.19	-0.36	-0.01	0.03		
	Cloud	0.01	-0.15	0.18	0.88		
MI + UC						2.00	0.09
	Moonlight index	-0.19	-0.36	-0.01	0.03		
	Understorey cover	0.01	-0.26	0.29	0.94		
MI × UC						2.02	0.09
	Moonlight index	-0.20	-0.38	-0.03	0.03		
	Understorey cover	0.03	-0.24	0.31	0.81		
	Moonlight Index:Understorey cover	0.12	-0.05	0.29	0.16		
Null						2.72	0.07

Supplementary Table S5. Heath Mouse (*Pseudomys shortridgei*). The positive estimate for season reflects lower activity in season 1 (2018/19) compared to season 2 (2019/20).

Model	Parameter	Estimate	Lower CL	Upper CL	P-value	Change in AICc	Akaike weight
MI + UC + C						0.00	0.32
	Moonlight index	-0.22	-0.30	-0.14	<0.001		
	Understorey cover	0.31	0.04	0.57	0.02		
	Cloud	0.15	0.07	0.24	<0.001		
MI + UC + C + S						0.47	0.25
	Moonlight index	-0.22	-0.30	-0.14	<0.001		
	Understorey cover	0.32	0.06	0.58	0.02		
	Cloud	0.14	0.06	0.23	<0.01		
	Season	0.10	-0.06	0.25	0.22		
MI × UC + C						1.07	0.18
	Moonlight index	-0.21	-0.29	-0.13	<0.001		
	Understorey cover	0.30	0.03	0.56	0.03		
	Moonlight index:Understorey cover	-0.04	-0.13	0.04	0.33		
	Cloud	0.15	0.07	0.23	<0.001		
MI × UC + C + S						1.53	0.15
	Moonlight index	-0.21	-0.29	-0.13	<0.001		
	Understorey cover	0.31	0.05	0.57	0.02		
	Moonlight index:Understorey cover	-0.04	-0.13	0.04	0.33		
	Cloud	0.14	0.06	0.22	<0.01		
	Season	0.10	-0.06	0.25	0.21		
Null						51.98	0.00

Supplementary Table S6. House Mouse (*Mus musculus*). The negative estimate for season reflects higher activity in season 1 (2018/19) compared to season 2 (2019/20).

Model	Parameter	Estimate	Lower CL	Upper CL	P-value	Change in AICc	Akaike weight
MI + UC + S						0.00	0.52
	Moonlight index	-0.40	-0.53	-0.27	<0.001		
	Understorey cover	-2.28	-2.91	-1.64	<0.001		
	Season	-1.81	-2.16	-1.45	<0.001		
MI × UC + S						1.85	0.21
	Moonlight index	-0.41	-0.55	-0.27	<0.001		
	Understorey cover	-2.29	-2.92	-1.65	<0.001		
	Moonlight index:Understorey cover	-0.04	-0.21	0.14	0.69		
	Season	-1.81	-2.16	-1.45	<0.001		
MI + UC + S + C						1.97	0.20
	Moonlight index	-0.40	-0.53	-0.26	<0.001		
	Understorey cover	-2.27	-2.90	-1.63	<0.001		
	Season	-1.81	-2.17	-1.45	<0.001		
	Cloud	0.01	-0.11	0.14	0.85		
Null						217.24	0.00

Supplementary Table S7. Yellow-footed Antechinus (*Antchinus flavipes*). The positive estimate for season reflects lower activity in season 1 (2018/19) compared to season 2 (2019/20).

Model	Parameter	Estimate	Lower CL	Upper CL	P-value	Change in AICc	Akaike weight
MI × UC + C						0.00	0.32
	Moonlight index	-0.25	-0.40	-0.09	<0.01		
	Understorey cover	0.36	0.04	0.68	0.03		
	Moonlight index:Understorey cover	-0.10	-0.23	0.03	0.14		
	Cloud	0.23	0.09	0.37	<0.01		
MI + UC + C						0.18	0.30
	Moonlight index	-0.30	-0.44	-0.16	<0.001		
	Understorey cover	0.39	0.07	0.71	0.02		
	Cloud	0.22	0.08	0.36	<0.01		
MI × UC + C + S						1.68	0.14
	Moonlight index	-0.25	-0.40	-0.09	<0.01		
	Understorey cover	0.36	0.04	0.69	0.03		
	Moonlight index:Understorey cover	-0.10	-0.23	0.03	0.14		
	Cloud	0.22	0.08	0.37	<0.01		
	Season	0.08	-0.18	0.34	0.57		
MI + UC + C + S						1.89	0.13
	Moonlight index	-0.30	-0.44	-0.16	<0.001		
	Understorey cover	0.39	0.07	0.71	0.02		
	Cloud	0.22	0.08	0.36	<0.01		
	Season	0.07	-0.19	0.33	0.59		
Null						27.04	0.00

Supplementary Table S8. Southern Brown Bandicoot (*Isoodon obesulus*). The positive estimate for season reflects lower activity in season 1 (2018/19) compared to season 2 (2019/20).

Model	Parameter	Estimate	Lower CL	Upper CL	P-value	Change in AICc	Akaike weight
MI + UC + S						0.00	0.25
	Moonlight index	-0.21	-0.41	-0.02	0.03		
	Understorey cover	0.84	0.44	1.23	<0.001		
	Season	0.36	-0.03	0.76	0.07		
MI × UC + S						0.94	0.16
	Moonlight index	-0.31	-0.59	-0.03	0.03		
	Understorey cover	0.88	0.47	1.29	<0.001		
	Moonlight index:Understorey cover	0.16	-0.15	0.47	0.32		
	Season	0.37	-0.03	0.76	0.07		
MI + UC						1.32	0.13
	Moonlight index	-0.21	-0.40	-0.02	0.04		
	Understorey cover	0.75	0.36	1.15	<0.001		
MI + UC + S + C						1.57	0.11
	Moonlight index	-0.23	-0.43	-0.03	0.02		
	Understorey cover	0.83	0.44	1.23	<0.001		
	Season	0.42	-0.01	0.84	0.05		
	Cloud	-0.07	-0.26	0.13	0.50		
Null						20.85	0.00

Supplementary Table S9. Feral cat (*Felis catus*).

Model	Parameter	Estimate	Lower CL	Upper CL	P-value	Change in AICc	Akaike weight
Null						0.00	0.18
UC + C						0.73	0.13
	Understorey cover	-0.29	-0.80	0.23	0.28		
	Cloud	0.40	-0.18	0.99	0.17		
UC						0.77	0.13
	Understorey cover	-0.29	-0.80	0.23	0.28		
MI + C						1.70	0.08
	Moonlight index	0.12	-0.35	0.59	0.62		
	Cloud	0.40	-0.17	0.98	0.17		
MI						1.81	0.07
	Moonlight index	0.11	-0.37	0.58	0.66		
MI × UC + C						3.84	0.03
	Moonlight index	0.17	-0.32	0.65	0.50		
	Understorey cover	-0.33	-0.86	0.21	0.24		
	Moonlight index:Understorey cover	0.20	-0.29	0.69	0.42		
	Cloud	0.40	-0.17	0.98	0.17		

Supplementary Table S10. Red fox (*Vulpes vulpes*). The positive estimate for season reflects lower activity in season 1 (2018/19) compared to season 2 (2019/20).

Model	Parameter	Estimate	Lower CL	Upper CL	P-value	Change in AICc	Akaike weight
UC + S						0.00	0.37
	Understorey cover	-0.23	-0.45	-0.01	0.04		
	Season	0.36	0.10	0.62	<0.01		
UC + S + C						1.64	0.17
	Understorey cover	-0.23	-0.45	-0.01	0.04		
	Season	0.37	0.11	0.63	<0.01		
	Cloud	-0.04	-0.17	0.09	0.54		
MI + UC +S						1.73	0.16
	Moonlight index	-0.03	-0.16	0.10	0.61		
	Understorey cover	-0.23	-0.45	-0.01	0.04		
	Season	0.36	0.10	0.62	<0.01		
MI × UC + S						3.66	0.06
	Moonlight index	-0.04	-0.17	0.09	0.57		
	Understorey cover	-0.23	-0.45	-0.01	0.04		
	Moonlight index:Understorey cover	-0.02	-0.15	0.11	0.78		
	Season	0.36	0.10	0.62	<0.01		
Null						7.74	0.01

Supplementary Table S11. Generalised linear mixed effects models built to test the predictions of the predation risk and habitat mediated predation risk hypotheses. ✓ represent hypotheses the model tests directly and ✕ represent hypotheses the model does not test directly. The first eight models contain the moonlight index (MI) but do not contain the interaction between the moonlight index and understorey cover (UC). These models provide a direct test of the predation risk (detectible negative relationship between species activity and MI) hypotheses. The following four models (models 9-12) contain the interaction between the moonlight index and understorey cover and provide a direct test of the habitat mediated predation risk hypothesis; a detectible interaction effect indicates that the response of species activity to MI depends on UC (the expectation is if the negative relationship between activity and MI is more rapid when UC is low compared to when it is high). Models 13-16 do not any of the hypotheses directly. They contain understorey cover but not the moonlight index and were included to contrast with the first eight MI models in a model selection process; strong support for the predation risk hypothesis will result in one of the first eight models being more parsimonious (e.g. smaller AICc, higher Akaike weight) than models 13-16. Similarly, strong support for the habitat mediated predation risk hypothesis will result in a model containing the MI by UC interaction (models 9-12) being more parsimonious than a model containing only the main effects of MI and UC (models 5-8). A null (intercept only) model was included as a reference point; support for either of the two hypotheses requires the most parsimonious model to be superior to the null model. Some models include season (S), cloud cover (C) or both to account for the variance attributable to these predictors.

Model	Predation risk	Habitat mediated predation risk
1. MI	✔	✕
2. MI + C	✔	✕
3. MI + S	✔	✕
4. MI + C + S	✔	✕
5. MI + UC	✔	✕
6. MI + UC + C	✔	✕
7. MI + UC + S	✔	✕
8. MI + UC + C + S	✔	✕
9. MI × UC	✕	✔
10. MI × UC + C	✕	✔
11. MI × UC + S	✕	✔
12. MI × UC + C + S	✕	✔
13. UC	✕	✕
14. UC + C	✕	✕
15. UC + S	✕	✕
16. UC + C + S	✕	✕
17. Null	Included for reference	

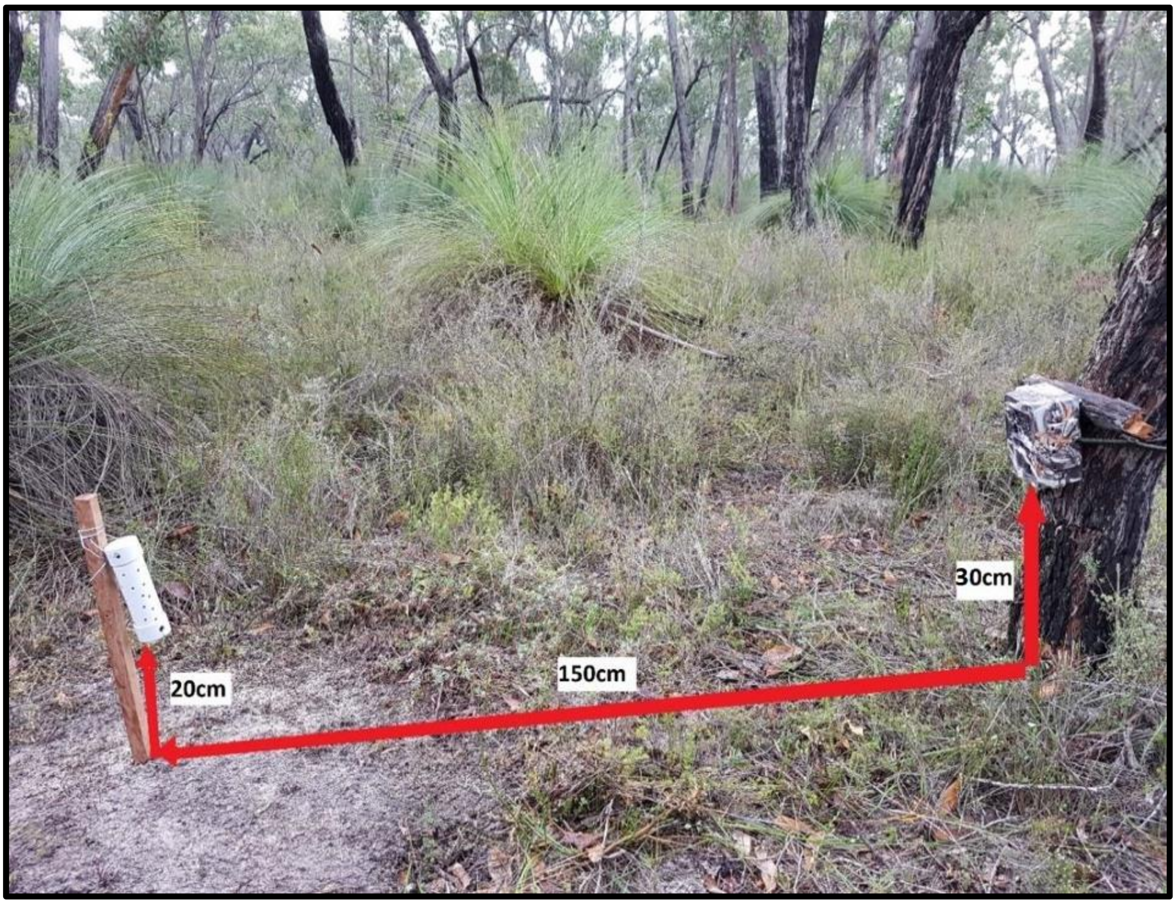

Supplementary Figure S1. Photograph showing how remote cameras were set up. Vegetation was clipped in a 2 m by 1 m area (50 cm on either side of the camera, between the camera and the bait station, and 50 cm beyond the bait station).
